# Associations between ^18^F-FDG-PET, DWI, and DCE Parameters in Patients with Head and Neck Squamous Cell Carcinoma Depend on Tumor Grading

**DOI:** 10.1155/2017/5369625

**Published:** 2017-10-19

**Authors:** Leonard Leifels, Sandra Purz, Patrick Stumpp, Stefan Schob, Hans Jonas Meyer, Thomas Kahn, Osama Sabri, Alexey Surov

**Affiliations:** ^1^Department of Diagnostic and Interventional Radiology, University Hospital of Leipzig, Liebigstrasse 20, 04103 Leipzig, Germany; ^2^Department of Nuclear Medicine, University Hospital of Leipzig, Liebigstraße 18, 04103 Leipzig, Germany; ^3^Division of Neuroradiology, University Hospital of Leipzig, Liebigstrasse 20, 04103 Leipzig, Germany

## Abstract

Our purpose was to analyze associations between positron emission tomography (PET), diffusion weighted imaging (DWI), and dynamic contrast-enhanced (DCE) imaging in patients with head and neck squamous cell carcinoma (HNSCC). The study involved 34 patients (9 women, 25 men, mean age: 56.7 ± 10.2 years). In all patients a simultaneous ^18^F-FDG-PET/MR was performed. DWI was obtained by using of an axial EPI sequence. Minimal ADC values (ADC_min_), mean ADC values (ADC_mean_), and maximal ADC values (ADC_max_) were estimated. DCE MRI was performed by using dynamic T1w DCE sequence. The following parameters were estimated: *K*_trans_, *V*_*e*_, and *K*_ep_. Spearman's correlation coefficient was used to analyze associations between investigated parameters. In overall sample, ADC_mean_ correlated significantly with *V*_*e*_ and *K*_trans_, ADC_min_ correlated with *V*_*e*_, and ADC_max_ correlated with *K*_trans_ and *V*_*e*_. SUV_mean_ tended to correlate slightly with *K*_trans_. In G1/2 tumors, only *K*_trans_ correlated well with ADC_max_ and SUV_mean_. In G3 tumors, *K*_trans_ correlated well with *K*_ep_ and *V*_*e*_. *V*_*e*_ showed significant correlations with ADC_mean_ and ADC_max_. *K*_trans_ correlated with ADC_max_. *K*_ep_ was higher in cancers with N2/3 stages. Tumor metabolism, water diffusion, and tumor perfusion have complex relationships in HNSCC. Furthermore, these associations depend on tumor grading. *K*_ep_ may predict lymphonodal metastasizing.

## 1. Introduction

Head and neck squamous cell carcinoma (HNSCC) is the most frequent malignancy of the upper aerodigestive tract in humans [1].

Contrast-enhanced computed tomography (CT) and magnetic resonance imaging (MRI) provide the mainstay of imaging for diagnosis, staging, and treatment response assessment in HNSCC [2]. Functional imaging such as positron emission tomography (PET), diffusion weighted imaging (DWI), and dynamic contrast-enhanced (DCE) MRI provide complementary information on the underlying biology such as metabolic activity, cellularity, vascularity, and oxygenation [2, 3].

It has been shown that HNSCC lesions had high standardized uptake values (SUV) and low apparent diffusion coefficient (ADC) values [4, 5]. Furthermore, malignant tumors showed also high perfusion parameters in comparison to benign lesions [6].

Some authors performed multiparametric investigation of HNSCC including ^18^F fluorodeoxyglucose PET (^18^F-FDG-PET), DWI, and DCE and attempted to characterize complexity of imaging findings reflecting tumor biology [3, 7, 8]. The reported data, however, were inconsistent. Some authors found significant correlations between analyzed parameters and, therefore, suggested complex interactions among tumor biologic characteristics [7–10]. Thereby, DWI, PET, and DCE parameters were reported to have similar potential to characterize HNSCC [10]. For example, Nakajo et al. showed that both SUV and ADC values correlated well together and could similarly predict disease-free survival or disease events in HNSCC [10].

However, in other studies, no significant correlations between these parameters were identified [11–13]. Therefore, it has been mentioned that parameters derived from PET, DWI, and DCE are independent biomarkers and complement one another [11–15].

This discrepancy of reported data questions the possibility of using multiparametric imaging findings as surrogate markers of tumor consistency in HNSCC.

The analysis of possible relationships between microcirculation, cellularity, and glucose metabolism has not only scientific importance but also clinical significance. As mentioned previously, an understanding of such complexities could expand the knowledge of tumor characteristics and have clinical implications such as in guidance for treatment planning, early prediction of treatment responses, and evaluation of treatment outcome [3].

The purpose of this study was to analyze possible associations between multiparametric imaging findings of simultaneous ^18^F-FDG-PET/MR including DWI and DCE in patients with HNSCC.

## 2. Materials and Methods

This prospective study was approved by the institutional review board of the University of Leipzig and all patients gave their written informed consent. All methods were performed in accordance with the relevant guidelines and regulations.

### 2.1. Patients

Overall, 34 patients with primary HNSCC of different localizations were involved in the study (Table 1). There were 9 (26%) women and 25 (74%) men with a mean age of 56.7 ± 10.2 years, range 33–77 years. At initial presentation, the tumors were localized in the tonsil (23.6%), followed by oropharynx (20.6%) and tongue (20.6%), hypopharynx (17.6%), larynx (14.6%), and epipharynx (2.9%). In most cases, high grade lesions (51.8%) were diagnosed. Furthermore, most frequently, the identified lesions were staged as T3 (29.4%) or T4 tumors (47.1%) with additional nodal (91.2%) metastases (Table 1).

### 2.2. Imaging

#### 2.2.1. Simultaneous PET/MR

In all patients a simultaneous ^18^F-FDG-PET/MR (Magnetom Biograph mMR-Biograph, Siemens Healthcare Sector, Erlangen, Germany) was performed from the upper thigh to the skull after a fasting period of at least 6 hours. Application of ^18^F-FDG was performed intravenously with a body weight-adapted dose (4 MBq/kg, range: 168–427 MBq, and mean ± SD: 279 ± 60 MBq). PET/MR image acquisition started on average 170 minutes (range 60–300 minutes) after ^18^F-FDG application. In 28/34 patients a PET/CT scan was performed prior to PET/MRI, which explains the later PET/MRI image acquisition time in these patients. For attenuation correction of the PET data a coronal 3D-encoded gradient-echo sequence (Dixon-VIBE) was used. For each tumor, maximum and mean SUV (SUV_max_; SUV_mean_) were determined.

PET images were analyzed by one nuclear medicine physician (S. P.) with 7 years of experience.

### 2.3. Image Interpretation

PET/MR datasets were evaluated by a board certified nuclear medicine and a board certified radiologist with substantial PET/MR experience in oncological image interpretation. PET/MR image analysis was performed on the dedicated workstation of Hermes Medical Solutions, Sweden.

**Figure 1:**
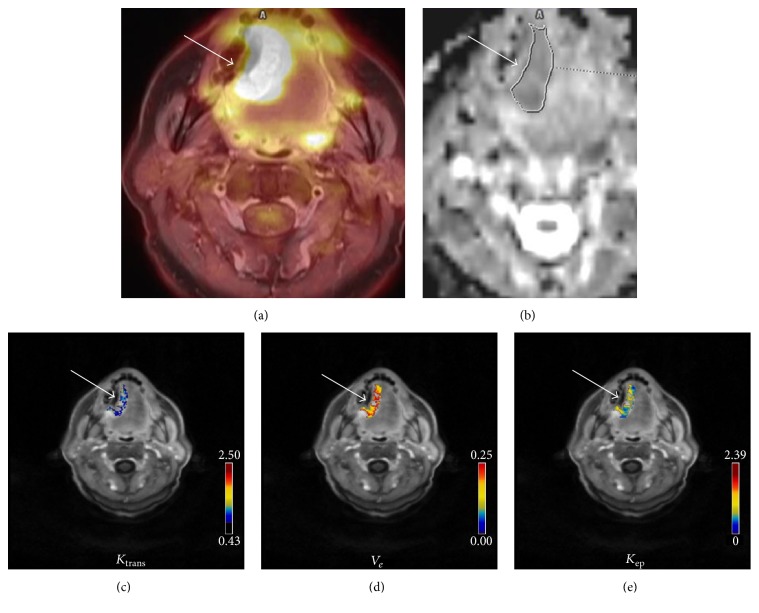
Imaging findings in a 58-year-old man with squamous cell carcinoma of the tongue (G1, T4 N2 M0). (a) ^18^F-FDG-PET imaging (fused image) showing a right sided large lesion of the tongue; SUV_max_ = 24.11. (b) ADC map. The ADC values (×10^−3^ mm^2^s^−1^) of the lesion are as follows: ADC_min_ = 0.68, ADC_mean_ = 0.97, and ADC_max_ = 2.1. (c–e) DCE imaging findings. Estimated DCE parameters are as follows: *K*_trans_ = 0.53 min^−1^ (c), *V*_*e*_ = 0.68% (d), and *K*_ep_ = 0.75 min^−1^ (e).

Tumor margins of the HNSCC were identified on MR images (T1-sequence) and a polygonal volume of interest (VOI) was placed in the fused PET/MR dataset (SUV_max_ threshold 40%) (Figure 1(a)).

### 2.4. DWI

DWI was obtained by using an axial EPI (echo planar imaging) sequence with *b*-values of 0 and 800 s/mm^2^ (TR/TE: 8620/73 ms, slice thickness 4 mm, and voxel size 3.2 × 2.6 × 4.0 mm). ADC maps were automatically generated by the implemented software. DWI images were analyzed by one radiologist (L. L., 2 years of general radiological experience). Polygonal regions of interest (ROI) were manually drawn on the ADC maps along the contours of the tumor on each slice (Figure 1(b)). In all lesions minimal ADC values (ADC_min_), mean ADC values (ADC_mean_), and maximal ADC values (ADC_max_) were estimated (Figure 1(b)).

### 2.5. DCE

In 31 patients, DCE MRI was performed by using dynamic T1w DCE sequence (TR/TE 2.47/0.97 ms, slice thickness 5 mm, flip angle 8°, and voxel size 1.2 × 1.0 × 5.0 mm) after intravenous application of contrast medium (0.1 mmol Gadobutrol per kg of body weight) (Gadovist®, Bayer Healthcare, Leverkusen, Germany) as reported previously [8, 15]. The acquired images were transferred to a software module for tissue perfusion estimation (Tissue 4D, Siemens Medical Systems, Erlangen, Germany). The software offers a population based approach for the arterial input function (AIF) and the best of three available AIF-options was chosen according to the result of the chi2-parameter, which serves as an error measure for the model fit [7, 8]. The AIF was scaled in relation to the gadolinium dose and modelled according to the biexponential model of Tofts and Kermode [16]. The following pharmacokinetic parameters [7, 8, 15] were automatically calculated for whole lesion in every case (Figures 1(c)–1(e)):

- *K*_trans_: volume transfer constant, which estimates the diffusion of contrast medium from the plasma through the vessel wall into the interstitial space, representing vessel permeability
- *V*_*e*_: volume of the extravascular extracellular leakage space (EES)
- *K*_ep_: parameter for diffusion of contrast medium from the EES back to the plasma. It is in close relation with *K*_trans_ and *V*_*e*_ and is calculated by the formula *k*_ep_ = *K*_trans_ × *V*_*e*_^−1^.

DCE images were analyzed by one radiologist (L. L., 2 years of general radiological experience).

### 2.6. Statistical Analysis

Statistical analysis and graphics creation were performed using SPSS 20 (IBM SPSS Statistics, Armonk, New York, USA). Collected data were evaluated by means of descriptive statistics (absolute and relative frequencies). Spearman's correlation coefficient (*p*) was used to analyze associations between investigated parameters. *P* values < 0.05 were taken to indicate statistical significance.

## 3. Results

A complete overview of the results including mean values, standard deviation, and ranges is shown in Table 2.

Сorrelation analysis identified the following: in overall sample, ADC_mean_ correlated significantly well with *V*_*e*_ (*P* = 0.0002) and slightly with *K*_trans_ (0.04), ADC_min_ correlated with *V*_*e*_ (*P* = 0.0007), and ADC_max_ correlated with *K*_trans_ (0.0032) and *V*_*e*_ (0.045) (Table 3). *K*_trans_ correlated well with *K*_ep_ (*P* = 0.0017) and *V*_*e*_ (*P* = 0.0002).

In addition, SUV_max_ tended to correlate slightly inversely with ADC_min_ (*P* = 0.08) and SUV_mean_ had a tendency to correlate with *K*_trans_ (*P* = 0.08).

On the next step, the estimated parameters were correlated in different tumor subgroups. In G1/2 tumors, *K*_trans_ correlated well with ADC_max_ and SUV_mean_ (Table 4). No other significant correlations were identified. SUV_max_ tended to correlate inversely with ADC_min_ (*P* = 0.09). *V*_*e*_ had a tendency to correlate with ADC_mean_ and ADC_min_ (in both cases, *P* = 0.08). In addition, DCE parameters did not correlate together.

However, in G3 tumors, *K*_trans_ correlated well with *K*_ep_ (*P* = 0.015) and *V*_*e*_ (*P* = 0.003) (Table 5). *V*_*e*_ showed significant strong correlations with ADC_mean_ (*P* = 0.0014) and ADC_min_ (*P* = 0.01). *K*_trans_ correlated moderately with ADC_max_ (*P* = 0.04). Finally, SUV values did not correlate with ADC and perfusion parameters.

No significant differences were identified in the analyzed parameters between poorly and moderately/well differentiated tumors (Table 6).

There were no significant differences of the analyzed parameters between several tumor stages (Tables 7(a)–7(c)). Only *K*_ep_ was higher in cancers with N2/3 stages versus N0/1 stages (Table 7(b)).

## 4. Discussion

Our study identified several significant associations between PET, DWI, and DCE parameters in primary HNSCC in a complex investigation.

The analysis of previous studies regarding multiparametric imaging findings in HNSCC shows that the reported results are controversial. This applies both comparisons of the investigated parameters in different tumor stages and correlation between the variables. For example, Fruehwald-Pallamar et al. analyzed sequentially acquired ^18^F-FDG-PET and MR images of 31 HNSCC patients and did not observe significant differences in ADC values and SUV_max_ between various T stages of the investigated tumors [11]. However, Kim et al. found that T3/4 tumors had higher SUV_max_ values than T1/2 lesions [17]. In addition, N positive tumors showed also higher SUV_max_ values [17]. According to Zhang et al., T3/4 tumors showed statistically significant higher ADC values in comparison to T1/2 lesions [18]. It has also been reported that DCE parameters correlated well with tumor stage in nasopharyngeal carcinoma [19].

In the present study, we also identified significant differences in analyzed parameters between several tumor stages. Firstly, advanced carcinomas had higher SUV_max_ values. However, there were no significant differences in other investigated parameters between T1/2 and T3/4 tumors. This finding suggests that advanced tumors have higher metabolic activity but not higher cell density or perfusion. Secondly, *K*_ep_ was higher in N2 tumors. Previously, strong correlations between *K*_ep_ and microvessel density in HNSCC were reported [15]. Therefore, our findings may indicate that tumor microvessel density might influence lymphatic metastatic spread in HNSCC.

To the best of our knowledge, previously, only two studies investigated associations between imaging findings and tumor grading in HNSCC [11, 12]. So, Choi et al. mentioned that poorly differentiated tumors had statistically significant lower ADC values and higher SUV values than G1/G2 tumors [12]. Other authors, however, reported that SUV and ADC values could not distinguish tumor stages [11]. Also in the present study no significant differences were identified between well/moderately and poorly differentiated carcinomas. Grading system of HNSCC includes the following parameters: degree of keratinization, nuclear pleomorphism, number of mitoses, pattern of invasion, and presence or absence of inflammatory infiltrates [20, 21]. However, this system does not include parameters, such as cell count, cell size, extracellular space, and microvessel density, which are known to influence water diffusion, glucose metabolism, and perfusion. This may explain our negative results regarding associations between tumor grading and multiparametric imaging findings.

According to previous reports, several parameters of tumor perfusion, diffusion, and glucose metabolism were associated together [7–10]. So Bisdas et al. identified significant correlations between SUV values and *V*_*e*_ (*p* = 0.42, *P* = 0.03) [7]. Furthermore, analyzed perfusions parameter (*K*_trans_, *V*_*e*_, and *K*_ep_) also correlated well together [7]. In the study of Nakajo et al., a statistically significant inverse correlation between SUV_max_ and ADC_mean_ (*p* = −0.566, *P* = 0.005) was found [10]. Additionally, according to Covello et al., ADC_mean_ correlated inversely with K_trans_ (*p* = −0.42, *P* = 0.04) [9].

However, other authors did not identify significant correlations between analyzed parameters [11–13]. For instance, Rasmussen et al. could not find significant associations between SUV and ADC values [13]. Similar results were also reported in other researches [11, 12, 14]. Furthermore, Han et al. detected no significant associations between DCE and glucose metabolism parameters in HNSCC [3].

It is still unclear why some authors found significant correlations between water diffusion, glucose metabolism, and perfusion parameters in HNSCC while others did not. Presumably, tumor heterogeneity may play a role here. For example, well, moderately, and poorly differentiated tumors might show also different associations of imaging parameters. In fact, our results confirmed this hypothesis.

In the present study, no significant correlations between different ADC parameters and SUV values were identified in overall sample. Furthermore, SUV_max_ tended to correlate slightly with *K*_trans_ and ADC_min_. All ADC parameters showed significant associations with *V*_*e*_. In addition, *K*_trans_ correlated slightly with ADC_mean_ and moderately with ADC_max_ and *K*_ep_ and well with *V*_*e*_.

Separate correlation analyses in the G1/2 and G3 tumors showed, however, other associations between the investigated parameters. As seen, perfusion parameters *K*_trans_, *V*_*e*_, and *K*_ep_ did not significantly correlate together in well and moderately differentiated tumors. However, they correlated well in high grade carcinomas. Additionally, *K*_trans_ correlated well with SUV_mean_ in G1/2 lesions but not in G3 tumors. Finally, *V*_*e*_ correlated well with ADC_mean_ and ADC_min_ in G3 tumors, but not in G1/2 lesions.

Our data suggest that tumor metabolism, cellularity, and perfusion show complex relationships in HNSCC. Furthermore, these associations depend on tumor grading. Previously, it has been shown that SUV and ADC values as well as perfusion parameters correlated with different histopathological features in HNSCC [14, 15]. We hypothesize on the basis of our findings that tumors with different grading may have also different relationships between cell count, stroma, and microvessel density, that is, different tumor architecture. Furthermore, our data suggest that tissue architecture plays a great role in tumor characteristic. Our findings may also explain controversial data of previous reports. Presumably, previous studies might contain well, moderately, and poorly differentiated lesions in several proportions that may result in different associations between the analyzed parameters.

In conclusion, multiparameter imaging provides information regarding tumor composition. Our study shows that tumor metabolism, water diffusion, and tumor perfusion have complex relationships in HNSCC. Furthermore, these associations depend on tumor grading. Perfusion parameter *K*_ep_ may predict lymphonodal metastasizing.

## Figures and Tables

**Table 1:** Localization and stage of the identified tumors.

	*n* (%)
*Diagnosis*	
Carcinoma of epipharynx	1 (2.9)
Carcinoma of oropharynx	7 (20.6)
Carcinoma of hypopharynx	6 (17.6)
Carcinoma of larynx	5 (14.7)
Carcinoma of tongue	7 (20.6)
Tonsillar carcinoma	8 (23.6)
*Tumor stage*	
T stage	
T1	1 (2.9)
T2	7 (20.6)
T3	10 (29.4)
T4	16 (47.1)
*N stage*	
N0	3 (8.8)
N1	6 (17.7)
N2	22 (64.7)
N3	3 (8.8)
*M stage*	
M0	30 (88.2)
M1	4 (11.8)
*Tumor grading*	
G1	1 (2.9)
G2	12 (35.3)
G3	21 (51.8)

**Table 2:** DWI, PET, and DCE parameters of HNSCC.

Parameters	M ± SD	Range
SUV_max_	19.01 ± 9.81	5.81–48.00
SUV_mean_	8.19 ± 3.55	3.76–17.70
ADC_mean_ × 10^−3^ mm^2^s^−1^	1.14 ± 0.21	0.78–1.68
ADC_min_ × 10^−3^ mm^2^s^−1^	0.71 ± 0.23	0.17–1.24
ADC_max_ × 10^−3^ mm^2^s^−1^	1.77 ± 0.30	1.35–2.39
*K*_trans_	0.20 ± 0.12	0.01–0.53
*K*_ep_	0.58 ± 0.69	0.16–3.37
*V*_*e*_	0.51 ± 0.18	0.05–0.79

**Table 3:** Correlations between DCE, DWI, and PET parameters in all tumors.

Parameters	SUV_max_	SUV_mean_	ADC_mean_	ADC_min_	ADC_max_	*K*_trans_	*K*_ep_	*V*_*e*_
SUV_max_	—	**p** = 0.86	*p* = −0.25	*p* = −0.30	*p* = 0.18	*p* = 0.09	*p* = 0.10	*p* = −0.07
		**P** < 0.0001	*P* = 0.15	*P* = 0.08	*P* = 0.32	*P* = 0.63	*P* = 0.59	*P* = 0.70
SUV_mean_		—	*p* = −0.08	*p* = −0.15	*p* = 0.22	*p* = 0.32	*p* = 0.26	*p* = 0.13
			*P* = 0.67	*P* = 0.39	*P* = 0.22	*P* = 0.08	*P* = 0.16	*P* = 0.49
ADC_mean_			—	**p** = 0.88	**p** = 0.54	**p** = 0.37	*p* = −0.10	**p** = 0.62
				**P** < 0.0001	**P** = 0.0009	**P** = 0.04	*P* = 0.847	**P** = 0.0002
ADC_min_				—	*p* = 0.27	*p* = 0.26	*p* = −0.08	**p** = 0.58
					*P* = 0.12	*P* = 0.16	*P* = 0.65	**P** = 0.0007
ADC_max_						**p** = 0.51	*p* = 0.12	**p** = 0.36
						**P** = 0.003	*P* = 0.52	**P** = 0.0445
*K*_trans_							**p** = 0.54	**p** = 0.62
							**P** = 0.0017	**P** = 0.0002
*K*_ep_						—	—	*p* = −0.12
								*P* = 0.51
*V*_*e*_								—

Significant correlations are highlighted in bold.

**Table 4:** Correlations between DCE, DWI, and PET parameters in G1 and 2 tumors.

Parameters	SUV_max_	SUV_mean_	ADC_mean_	ADC_min_	ADC_max_	*K*_trans_	*K*_ep_	*V*_*e*_
SUV_max_	—	**p** = 0.74	*p* = −0.47	*p* = −0.49	*p* = 0.10	*p* = 0.27	*p* = 0.16	*p* = 0.14
		**P** = 0.0041	*P* = 0.10	*P* = 0.09	*P* = 0.75	*P* = 0.42	*P* = 0.63	*P* = 0.69
SUV_mean_		—	*p* = −0.23	*p* = −0.29	*p* = 0.31	**p** = ****0.65	*p* = 0.24	*p* = 0.34
			*P* = 0.45	*P* = 0.34	*P* = 0.30	**P** = 0.03	*P* = 0.48	*P* = 0.31
ADC_mean_			—	**p** = 0.68	**p** = 0.56	*p* = 0.44	*p* = −0.10	*p* = 0.55
				**P** = 0.01	**P** = 0.046	*P* = 0.18	*P* = 0.77	*P* = 0.08
ADC_min_				—	*p* = −0.08	*p* = 0.22	*p* = 0.11	*p* = 0.55
					*P* = 0.79	*P* = 0.52	*P* = 0.75	*P* = 0.08
ADC_max_						**p** = 0.65	*p* = 0.09	*p* = 0.25
						**P** = 0.03	*P* = 0.79	*P* = 0.45
*K*_trans_							*p* = 0.36	*p* = 0.500
							*P* = 0.27	*P* = 0.12
*K*_ep_								*p* = −0.37
								*P* = 0.26
*V*_*e*_								

Significant correlations are highlighted in bold.

**Table 5:** Correlations between DCE, DWI, and PET parameters in G3 tumors.

Parameters	SUV_max_	SUV_mean_	ADC_mean_	ADC_min_	ADC_max_	*K*_trans_	*K*_ep_	*V*_*e*_
SUV_max_	—	**p** = 0.85	*p* = −0.25	*p* = −0.26	*p* = 0.11	*p* = 0.02	*p* = 0.15	*p* = −0.24
		**P** < 0.0001	*P* = 0.28	*P* = 0.27	*P* = 0.65	*P* = 0.95	*P* = 0.54	*P* = 0.33
SUV_mean_		—	*p* = −0.11	*p* = −0.09	*p* = 0.05	*p* = 0.10	*p* = 0.34	*p* = −0.12
			*P* = 0.63	*P* = 0.71	*P* = 0.82	*P* = 0.69	*P* = 0.16	*P* = 0.63
ADC_mean_			—	**p** = 0.94	**p** = 0.60	*p* = 0.33	*p* = −0.15	**p** = 0.68
				**P** < 0.0001	**P** = 0.005	*P* = 0.17	*P* = 0.55	**P** = 0.0014
ADC_min_				—	**p** = 0.47	*p* = 0.18	*p* = −0.16	**p** = 0.57
					**P** = 0.04	*P* = 0.45	*P* = 0.52	**P** = 0.01
ADC_max_					—	**p** = 0.48	*p* = 0.16	*p* = 0.37
						**P** = 0.04	*P* = 0.51	*P* = 0.12
*K*_trans_						—	**p** = 0.55	**p** = 0.65
							**P** = 0.015	**P** = 0.003
*K*_ep_							—	*p* = −0.06
								*P* = 0.79
*V*_*e*_								—

Significant correlations are highlighted in bold.

**Table 6:** Comparison of PET and DWI values between different tumor grades.

Parameters	G1 + 2	G3 + 4	Mann–Whitney *U*
	Mean ± SD	Mean ± SD	*p* values
SUV_max_	21.11 ± 8.37	17.79 ± 9.86	0.20
SUV_mean_	8.92 ± 3.92	7.75 ± 3.40	0.28
ADC_min_ × 10^−3^ mm^2^s^−1^	0.74 ± 0.15	0.69 ± 0.28	0.65
ADC_mean_ × 10^−3^ mm^2^s^−1^	1.16 ± 0.14	1.13 ± 0.25	0.47
ADC_max_ × 10^−3^ mm^2^s^−1^	1.75 ± 0.25	1.79 ± 0.34	0.96
*K*_trans_	0.20 ± 0.13	0.20± 0.12	0.93
*K*_ep_	0.39 ± 0.16	0.70 ± 0.85	0.22
*V*_*e*_	0.55 ± 0.19	0.49 ± 0.17	0.37

**Table:** (a) Comparison of PET and DWI values between different tumor T stages

Parameters	T1/2 (mean ± SD)	T3/4 (mean ± SD)	Mann–Whitney *U*
			(*p* values)
SUV_max_	14.98 ± 7.88	20.25 ± 9.34	0.19
SUV_mean_	6.46 ± 1.60	8.73 ± 3.83	0.17
ADC_min_ × 10^−3^ mm^2^s^−1^	0.64 ± 0.23	0.73 ± 0.23	0.41
ADC_mean_ × 10^−3^ mm^2^s^−1^	1.09 ± 0.17	1.16 ± 0.21	0.56
ADC_max_ × 10^−3^ mm^2^s^−1^	1.73 ± 0.33	1.78 ± 0.30	0.58
*K*_trans_	0.17 ± 0.13	0.21 ± 0.11	0.27
*K*_ep_	1.05 ± 1.25	0.42 ± 0.17	0.37
*V*_*e*_	0.41 ± 0.22	0.55 ± 0.15	0.25

**Table:** (b) Comparison of PET and DWI values between different tumor N stages

Parameters	N 0/1 (mean ± SD)	N 2 (mean ± SD)	Mann–Whitney *U*
			(*p* values)
SUV_max_	19.72 ± 7.99	18.75 ± 9.72	0.78
SUV_mean_	8.50 ± 3.88	8.08 ± 3.50	0.97
ADC_min_ × 10^−3^ mm^2^s^−1^	0.78 ± 0.24	0.69 ± 023	0.75
ADC_mean_ × 10^−3^ mm^2^s^−1^	1.18 ± 0.24	1.13 ± 0.20	0.64
ADC_max_ × 10^−3^ mm^2^s^−1^	1.78 ± 0.29	1.77 ± 0.31	0.88
*K*_trans_	0.16 ± 0.08	0.22 ± 0.13	0.25
*K*_ep_	0.32 ± 0.11	0.68 ± 0.79	**0.0477**
*V*_*e*_	0.52 ± 0.18	0.50 ± 0.18	0.88

**Table:** (c) Comparison of PET and DWI values between different tumor M stages

Parameters	M0 (mean ± SD)	M1 (mean ± SD)	Mann–Whitney *U*
			(*p* values)
SUV_max_	18.98 ± 9.14	16.49 ± 10.06	0.56
SUV_mean_	8.01 ± 3.40	6.87 ± 1.50	0.60
ADC_min_	0.68 ± 0.22	0.89 ± 0.28	0.21
ADC_mean_	1.12 ± 0.19	1.28 ± 0.30	0.33
ADC_max_	1.76 ± 0.30	1.89 ± 0.37	0.46
*K*_trans_	0.21 ± 0.12	0.14 ± 0.08	0.47
*K*_ep_	0.62 ± 0.72	0.28 ± 0.17	0.16
*V*_*e*_	0.50 ± 0.19	0.55 ± 0.10	0.78

## References

[1] Braakhuis B. J. M., Leemans C. R., Visser O. (2014). Incidence and survival trends of head and neck squamous cell carcinoma in the Netherlands between 1989 and 2011. *Oral Oncology*.

[2] Powell C., Schmidt M., Borri M. (2013). Changes in functional imaging parameters following induction chemotherapy have important implications for individualised patient-based treatment regimens for advanced head and neck cancer. *Radiotherapy & Oncology*.

[3] Han M., Kim S. Y. O., Lee S. J. I., Choi J. W. O. (2015). The correlations between mri perfusion, diffusion parameters, and ^18^F-FDG PET metabolic parameters in primary head-and-neck cancer: a cross-sectional analysis in single institute. *Medicine*.

[4] Wang J., Takashima S., Takayama F. (2001). Head and neck lesions: characterization with diffusion-weighted echo-planar MR imaging. *Radiology*.

[5] Khan N., Oriuchi N., Ninomiya H., Higuchi T., Kamada H., Endo K. (2004). Positron emission tomographic imaging with 11C-choline in differential diagnosis of head and neck tumors: Comparison with 18F-FDG PET. *Annals of Nuclear Medicine*.

[6] Noij D. P., De Jong M. C., Mulders L. G. M. (2015). Contrast-enhanced perfusion magnetic resonance imaging for head and neck squamous cell carcinoma: a systematic review. *Oral Oncology*.

[7] Bisdas S., Seitz O., Middendorp M. (2010). An exploratory pilot study into the association between microcirculatory parameters derived by MRI-based pharmacokinetic analysis and glucose utilization estimated by PET-CT imaging in head and neck cancer. *European Radiology*.

[8] Gawlitza M., Purz S., Kubiessa K. (2015). In vivo correlation of glucose metabolism, cell density and microcirculatory parameters in patients with head and neck cancer: initial results using simultaneous PET/MRI. *PLoS ONE*.

[9] Covello M., Cavaliere C., Aiello M. (2015). Simultaneous PET/MR head-neck cancer imaging: preliminary clinical experience and multiparametric evaluation. *European Journal of Radiology*.

[10] Nakajo M., Nakajo M., Kajiya Y. (2012). FDG PET/CT and diffusion-weighted imaging of head and neck squamous cell carcinoma: comparison of prognostic significance between primary tumor standardized uptake value and apparent diffusion coefficient. *Clinical Nuclear Medicine*.

[11] Fruehwald-Pallamar J., Czerny C., Mayerhoefer M. E. (2011). Functional imaging in head and neck squamous cell carcinoma: correlation of PET/CT and diffusion-weighted imaging at 3 Tesla. *European Journal of Nuclear Medicine and Molecular Imaging*.

[12] Choi S. H., Paeng J. C., Sohn C.-H. (2011). Correlation of ^18^F-FDG uptake with apparent diffusion coefficient ratio measured on standard and high *b* value diffusion MRI in head and neck cancer. *Journal of Nuclear Medicine*.

[13] Rasmussen J. H., Nørgaard M., Hansen A. E. (2017). Feasibility of multiparametric imaging with PET/MR in head and neck Squamous cell carcinoma. *Journal of Nuclear Medicine*.

[14] Surov A., Stumpp P., Meyer H. J. (2016). Simultaneous ^18^F-FDG-PET/MRI: associations between diffusion, glucose metabolism and histopathological parameters in patients with head and neck squamous cell carcinoma. *Oral Oncology*.

[15] Surov A., Meyer H. J., Gawlitza M. (2017). Correlations between DCE MRI and histopathological parameters in head and neck squamous cell carcinoma. *Translational Oncology*.

[16] Tofts P. S., Kermode A. G. (1991). Measurement of the blood‐brain barrier permeability and leakage space using dynamic MR imaging. 1. Fundamental concepts. *Magnetic Resonance in Medicine*.

[17] Kim S. Y., Roh J.-L., Kim J. S. (2008). Utility of FDG PET in patients with squamous cell carcinomas of the oral cavity. *European Journal of Surgical Oncology*.

[18] Zhang Y., Liu X., Zhang Y. (2015). Prognostic value of the primary lesion apparent diffusion coefficient (ADC) in nasopharyngeal carcinoma: a retrospective study of 541 cases. *Scientific Reports*.

[19] Zheng D., Chen Y., Chen Y. (2014). Dynamic contrast-enhanced MRI of nasopharyngeal carcinoma: a preliminary study of the correlations between quantitative parameters and clinical stage. *Journal of Magnetic Resonance Imaging*.

[20] Sawazaki-Calone I., Rangel A. L. C. A., Bueno A. G. (2015). The prognostic value of histopathological grading systems in oral squamous cell carcinomas. *Oral Diseases*.

[21] Anneroth G., Hansen L. S. (1984). A methodologic study of histologic classification and grading of malignancy in oral squamous cell carcinoma. *European Journal of Oral Sciences*.

